# Use of resuscitation promoting factors to screen for tuberculosis infection in household-exposed children in The Gambia

**DOI:** 10.1186/s12879-020-05194-1

**Published:** 2020-07-02

**Authors:** W. van Loon, M. P. Gomez, D. Jobe, K. L. M. C. Franken, T. H. M. Ottenhoff, M. Coninx, L. Kestens, J. S. Sutherland, B. Kampmann, L. D. Tientcheu

**Affiliations:** 1 Institute of Tropical Medicine and International Health, Charité-University Medicine Berlin, Berlin, Germany; 2 Vaccines and Immunity Theme, MRC Unit The Gambia at London School of Hygiene & Tropical Medicine, Fajara, The Gambia; 3 Department of Infectious Diseases, Leiden University Medical Center, Leiden, The Netherlands; 4 Immunology Department, Institute of Tropical Medicine Antwerp, Antwerp, Belgium; 5 Faculty of Pharmaceutical, Biomedical and Veterinary Sciences, University of Antwerp, Antwerp, Belgium; 6 The Vaccine Centre, Faculty of Infectious & Tropical Diseases, London School of Hygiene & Tropical Medicine, London, UK; 7 Department of Biochemistry, Faculty of Science, University of Yaoundé 1, Yaoundé, Cameroon; 8 Department of Infection Biology, Faculty of Infectious and Tropical Diseases, London School of Hygiene & Tropical Medicine, London, UK

**Keywords:** Pediatric tuberculosis, IGRA, RpfB, RpfD, Immunology, Diagnostics

## Abstract

**Background:**

Interferon-γ release assays (IGRA) with Resuscitation promoting factor (Rpf) proteins enhanced tuberculosis (TB) screening and diagnosis in adults but have not been evaluated in children. Children often develop paucibacillary TB and their immune response differs from that of adults, which together affect TB disease diagnostics and immunodiagnostics. We assessed the ability of Rpf to identify infection among household TB-exposed children in The Gambia and investigated their ability to discriminate *Mycobacterium tuberculosis* complex (MTBC) infection from active TB disease in children.

**Methods:**

Detailed clinical investigations were done on 93 household TB-exposed Gambian children and a tuberculin skin test (TST) was administered to asymptomatic children. Venous blood was collected for overnight stimulation with ESAT-6/CFP-10-fusion protein (EC), purified protein derivative and RpfA, B, C, D and E. Interferon gamma (IFN-γ) production was measured by ELISA in supernatants and corrected for the background level. Infection status was defined by IGRA with EC and TB disease by mycobacterial confirmation and/or clinical diagnosis. We compared IFN-γ levels between infected and uninfected children and between infected and TB diseased children using a binomial logistic regression model while correcting for age and sex. A Receiver Operating Characteristics analysis was done to find the best cut-off for IFN-γ level and calculate sensitivity and specificity.

**Results:**

Interferon gamma production was significantly higher in infected (IGRA+, *n* = 45) than in uninfected (IGRA-, *n* = 20) children after stimulation with RpfA, B, C, and D (*P* = 0.03; 0.007; 0.03 and 0.003, respectively). Using RpfB and D-specific IFN-γ cut-offs (33.9 pg/mL and 67.0 pg/mL), infection was classified with a sensitivity-specificity combination of 73–92% and 77–72% respectively, which was similar to and better than 65–75% for TST. Moreover, IFN-γ production was higher in infected than in TB diseased children (*n* = 28, 5 bacteriologically confirmed, 23 clinically diagnosed), following RpfB and D stimulation (*P* = 0.02 and 0.03, respectively).

**Conclusion:**

RpfB and RpfD show promising results for childhood MTBC infection screening, and both performed similar to and better than the TST in our study population. Additionally, both antigens appear to discriminate between infection and disease in children and thus warrant further investigation as screening and diagnostic antigens for childhood TB.

## Background

About 10% of new and relapse tuberculosis (TB) cases occur in children under 15 years of age; accounting for at least 1 million cases a year [1]. Identifying and treating TB in children forms an essential part of TB control but detecting infection and diagnosing paediatric TB are challenging. Principally due to its paucibacillary nature, microbiological diagnosis of TB disease in children is insensitive. Hence, diagnosis is mainly based on clinical symptoms, which resemble those of other respiratory diseases [2]. Screening for *Mycobacterium tuberculosis complex* (MTBC) infection is done by the widely implemented tuberculin skin test (TST) or the interferon gamma (IFN-γ) release assay (IGRA), both assessing the host’s cell mediated immune response to tuberculous antigens [3]. Both tests have their limitations [4]. The TST –employing purified protein derivative (PPD) as antigen—has a low specificity due to cross reactivity with Bacillus Calmette-Guérin (BCG) vaccination and exposure to non-pathogenic environmental mycobacteria [5]. The IGRA –employing antigens 6 kDa early secretory antigenic target (ESAT-6) and 10 kDa culture filtrate (CFP-10) (EC) as peptide pool or as fusion protein—is less sensitive for detection of *M. africanum* (*Maf*) infection compared to the classical *M. tuberculosis (Mtb)* sensu stricto strains infection [6]; particularly important for countries like The Gambia where up to half of MTBC infections are caused by *Maf* strains [7]. Both the TST and IGRA cannot distinguish between infection and TB disease or individuals with high risk of progressing towards TB disease [8, 9]. These separations are important, as patients need to receive timely and appropriate treatment.

Evaluating new antigens for stimulation assays might solve the issues with sensitivity and specificity for MTBC infection screening. Antigens of recent interest are Resuscitation promoting factors (Rpf); secreted bacterial proteins initially characterized by their capacity to resuscitate nonreplicating cells in vitro and in vivo through lysozyme and peptidoglycan hydrolase activities [10]. Resuscitation promoting factors are specifically secreted by mycobacteria that shift from a dormant to their active replicating stage, in which they cause symptomatic disease [11, 12]. Five homologous Rpf genes (Rv0867c (RpfA); Rv1009 (RpfB); Rv1884c (RpfC); Rv2389c (RpfD); Rv2450c (RpfE)) have been identified in the genome of several mycobacteria, including *Mtb*, *Maf* and BCG [12, 13]. Resuscitation promoting factor A-, D- and E-specific IFN-γ responses in adults differ between infected and TB diseased individuals [14, 15]. Moreover, Huang et al found that the IFN-γ response to RpfA and D was associated with different levels of TB exposure and that it could possibly predict progression towards active disease in adults [16].

To the best of our knowledge, Rpf-specific IFN-γ responses have not previously been evaluated in children and could potentially be employed to improve childhood TB screening and diagnostics. We assessed the ability of Rpf to detect MTBC infection and to discriminate infection from TB disease among household-exposed children in The Gambia.

## Methods

### Study population and ethics

This study was approved by the MRC/Gambian government joint ethics committee. From January to December 2017, children aged below 15 years who were permanent household contacts of an adult smear-positive index TB case were recruited into the study. Written informed consent was obtained from the parents/guardians of the children prior to inclusion into the study.

### Clinical examination

Symptom screening was performed with a standard questionnaire including cough, fatigue, haemoptysis, weight loss, fever, neck swelling, night sweats and wheezing. Participants were considered symptomatic if persistent unremitting cough of ≥2 weeks duration was reported, with at least one other symptom as described before [17]. A TST was performed on all asymptomatic children, using the Mantoux method (2 tuberculin units, PPD RT23 *Statens Serum Institut*, Denmark), and induration size was read 48–72 h later. A skin induration of ≥10 mm measured transversely was considered TST positive (TST+), in line with WHO recommendations [18]. To save the scarce and expensive PPD antigens for children that needed it most, children that were considered symptomatic did not receive a TST. All children that were either TST+ or symptomatic underwent detailed clinical evaluation in the MRC clinic, including HIV testing and a chest X-ray as described before [17].

### Sample collection

Sputum was collected from all participants that were symptomatic and/or had an abnormal chest X-ray, either spontaneously or induced in children who were unable to expectorate. Samples were used for sputum microscopy, MTB/RIF assay (Gene*Xpert, Cepheid*) and mycobacterial liquid culture in the *MGIT BACTEC* instrument (*Becton-Dickinson*). All culture positive samples were genotyped using the spoligotyping method. For each adult TB index case up to 3 TST- and 3 TST+ children living within their household were included in this study. Venous blood (5 mL) was collected in a heparinized tube and stimulated with antigens within 4 h of collection. The laboratory team was blinded to all clinical information of the participants.

### Rpf antigens

Recombinant RpfA-E were kindly provided by Leiden University Medical Centre, The Netherlands and produced as previously described [19]. The antigens were freeze-dried and shipped at room temperature. The same batch of antigens was used throughout the study.

### Whole blood stimulation assay and IGRA

Heparinized whole blood was stimulated on a U-bottom 96-well plate in duplicate with and without stimulant. According to blood volume available, the antigens included RpfA-E (final concentration 10 μg/mL) [16], PPD (final concentration 10 μg/mL, RT23, *Statens Serum Institute*, Denmark), ESAT-6/CFP-10 (EC)-fusion protein (final concentration 10 μg/mL) [3, 20–22], Phytohemagglutinin Antigen-L (PHA-L, final concentration 10 μg/mL, *Sigma Aldrich*, USA) in RPMI-1640 medium (complemented with Penicillin and Streptomycin, final concentration 100 U/mL Penicillin, 100 μg/mL Streptomycin, *Gibco Invitrogen*, USA) to a final volume of 100 μl/well. Plates were incubated for 18–22 h at 37 °C, 5% CO_2_, followed by harvesting and freezing the supernatants at − 20 °C. The IFN-γ concentration in the supernatants was quantified with enzyme-linked immunosorbent assay (ELISA) (in-house ELISA, performed as described before [21]) and the optical density (OD) was read at 450 nm (*Softmax Pro* plate reader). Concentrations were calculated with a four-parameter curve fit (*Softmax Pro* software). The IGRA cut-off was set to 124.2 pg/mL, derived from the IFN-γ concentration mean of all unstimulated samples plus two times standard deviation [21, 23]. EC-specific IFN-γ levels above the cut-off were considered IGRA positive (IGRA+).

### Definition of MTBC infection and TB disease

All participants that were IGRA+ were defined as “MTBC infected” and all IGRA negative (IGRA-) participants as “MTBC uninfected”. “TB disease” was defined by mycobacterial confirmation and/or clinical diagnosis (i.e., suggestive appearance on chest radiograph, no response to empirical broad-spectrum antibiotics or favourable response to anti-tuberculous therapy), as described before [21].

### Data analysis

Categorical data were reported as frequency and proportions. Medians and interquartile ranges (IQRs) were calculated for non-normally distributed continuous data. IFN-γ levels were corrected to antigen specific responses by subtracting the IFN-γ level in the unstimulated control (i.e.*,* baseline IFN-γ level). Continuous TST (in mm) and IGRA (in IFN-γ concentration) results were compared using Spearman’s rank correlation. Binomial TST, IGRA and TB disease status results were compared with age (in years) using a Mann Whitney U-test and with sex using a two-tailed Fisher’s test. For every antigen, ln (IFN-γ) (natural logarithm transformation) was compared between the different infection/disease groups with a binomial logistic regression model while correcting for age and sex. The log-transformation was done to minimise skewed distributions of IFN-y concentrations. Significance level was *p* < 0.05. A Bonferroni correction was used to correct for multiple testing of 5 antigens of interest. *P*-values < 0.01 were considered highly significant (0.05/5 = 0.01). Antigens that induced highly significant differences were further investigated: a Receiver Operating Characteristic (ROC) curve was produced for varying infection/disease classifications with the Rpf-specific IFN-γ values. The area under the curve (AUC) of the ROC plots was calculated and an ideal cut-off was chosen using the Youden Index [24]. *R* version 3.2.3 was used for all analysis, specifically package *stats* for logistic regression analysis and package *ROCR* for ROC-curve analysis.

## Results

### Study participant demographics, TST screening and TB disease diagnosis

**Fig. 1:**
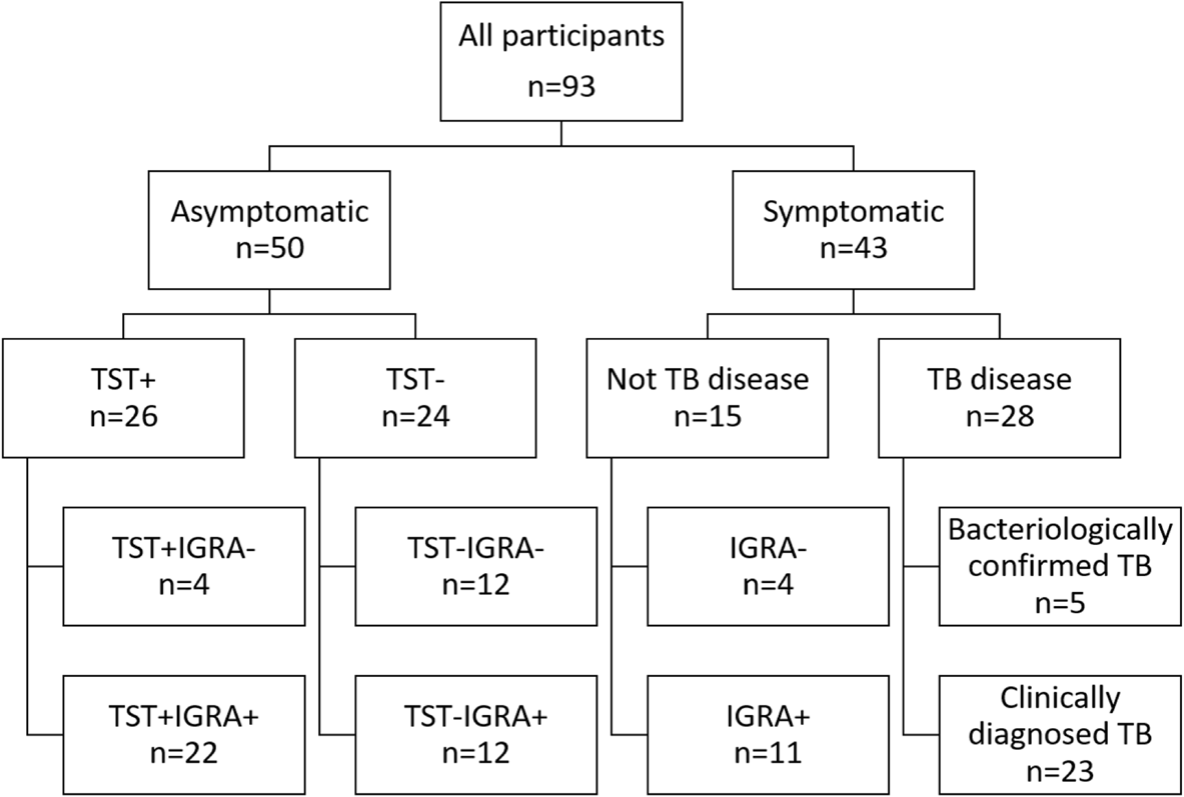
Flow diagram of all participants per screening category included in the study. TST: tuberculin skin test; IGRA: interferon gamma release assay; TB: tuberculosis; EC: ESAT-6/CFP-10 fusion protein

A total of 93 children all tested HIV-negative were included in this study with a median (IQR) age of 8.0 (4.7–10.9) years and 47 (50.5%) were female. Forty-three (43; 46.2%) children were symptomatic with a median (IQR) age of 9.7 (6.2–12.0). A BCG scar was present in 61 out of 66 (92.4%) assessed children. TB disease was diagnosed in 28 (30.1%) children (5 cases were bacteriologically confirmed TB all of the *M. tuberculosis* Euro-American lineage also called *Mtb*-lineage 4 and clinically diagnosed TB were 23 cases) (Fig. 1). The remaining 15 children were deemed to have other respiratory diseases. Fifty children (all asymptomatic) received the Mantoux test (tuberculin skin test; TST) and 26 (52.0%) were TST+.

### IGRA results and MTBC infection

To define MTBC infection status of the study participants, the EC-fusion protein IGRA result was taken as gold standard, with IFN-γ cut-off 124.2 pg/mL. Overall, EC-fusion protein induced a high IFN-γ response in all study participants (median (IQR) 275.5 (74.7–1435.0) pg/mL). Among the 65 children without TB disease (asymptomatic or symptomatic), 45 (70.3%) were IGRA+.

### Comparison of TST and IGRA results, and age within the subgroups

Among the 50 children who received a TST, 22 (44.0%) were TST + IGRA+, 12 (24.0%) were TST-IGRA- and 16 children had discordant results (Fig. 1). Tuberculin skin test and IGRA results (in mm skin induration and IFN-γ levels, respectively) were correlated (Spearman’s ρ 0.4; *P* < 0.001) [see Additional file 1, Supplementary Figure 1]. Tuberculin skin test+ children were older than TST- children (median (IQR) 7.5 (5.0–8.8) years versus 4.0 (3.0–7.3) years; *P* = 0.05) [see Additional file 1, Supplementary Figure 2]. There was a borderline age difference between IGRA+ (infected) and IGRA- (uninfected) children (median (IQR) 8.0 (5.0–10.0) years versus 4.5 (3.0–8.0) years; *P* = 0.06) [see Additional file 1, Supplementary Figure 3]. TB diseased children were significantly older than children without TB disease (irrespective of infection status) (median (IQR) 9.7 (6.0–12.2) years versus 7.0 (4.0–9.0) years, *P* = 0.02). There was no difference in age between children with bacteriologically confirmed and clinically diagnosed TB disease. We observed a trend of TB disease and infection in older children compared to younger children, which aligns with the slow progression to nature of MTBC infection. Resuscitation promoting factor-specific IFN-γ responses did not differ between children age below 5 years and above 5 years, and there was no correlation between Rpf-specific IFN-γ levels and age in years (data not shown).

### IFN-γ response to RpfA-D in MTBC infected compared to uninfected children without TB disease

**Fig. 2:**
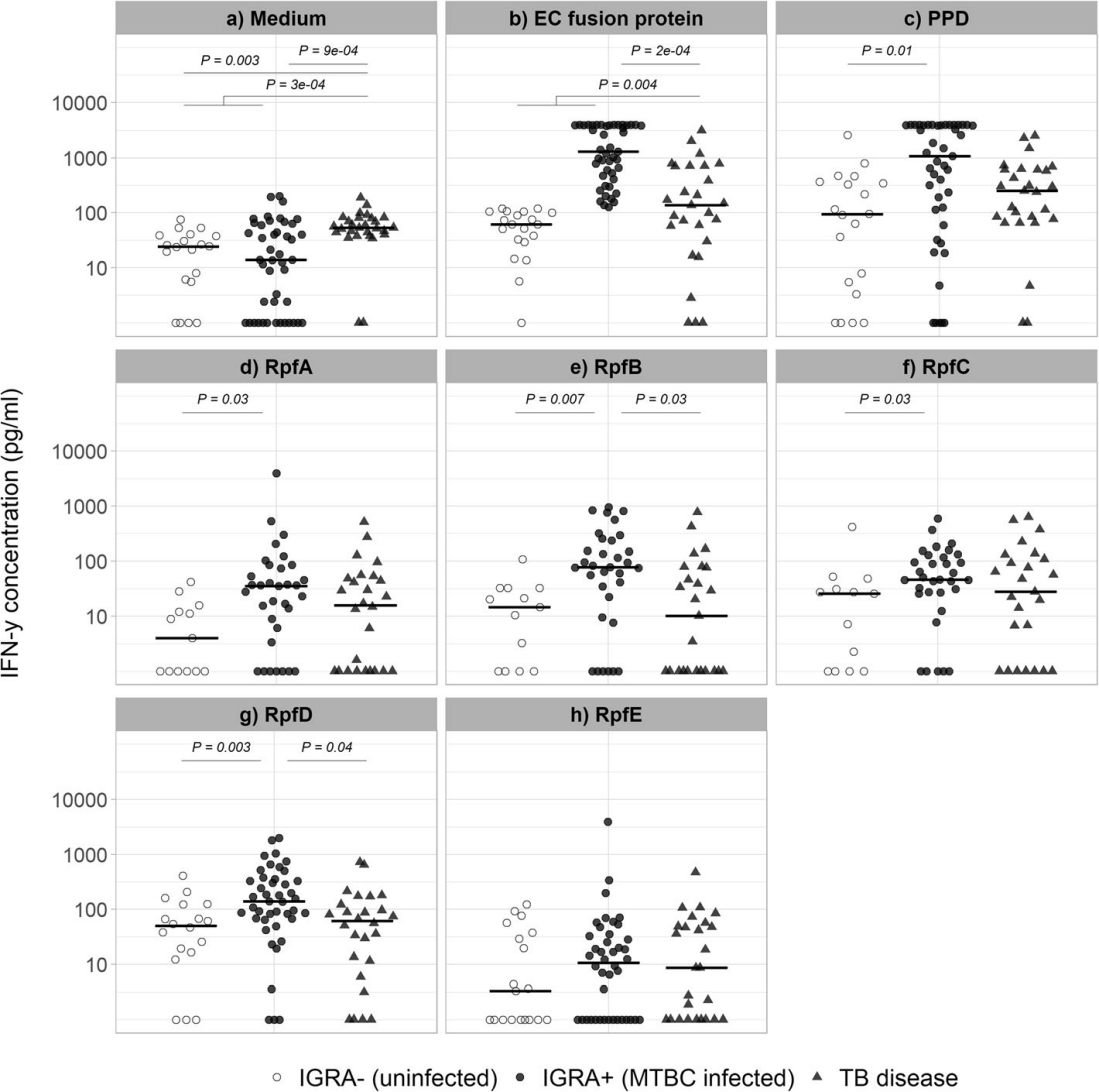
IFN-γ response after ex vivo whole blood antigen stimulation. The first panel (**a**) “medium” depicts the results of the unstimulated assays, i.e., the background level. The other panels (**b**-**h**) represent the respective antigen-stimulated assays. Solid lines: median IFN- γ values per infection category. IFN-γ concentrations were corrected for the background signal (value of unstimulated IFN-γ level subtracted), except for the results in the first panel “medium”. All values above 4000 were set to 4000 pg/mL. *P*-values below 0.05 indicated. P-values were derived from logistic regression models including possible confounders age and sex, after log-transformation of the IFN-γ variable. IGRA: IFN-γ release assay; EC: Esat-6/CFP-10; PPD: Purified protein derivative; Rpf: Resuscitation promoting factor

Whole blood stimulation with Rpf induced significantly higher IFN-γ responses in the infected (IGRA+) compared to the uninfected (IGRA-) children among those without TB disease. The medium (IQR) IFN-γ level of infected versus uninfected children was 34.6 (5.2–73.0) pg/mL versus 3.0 (0.0–11.1) pg/mL (*P* = 0.03) for RpfA, 77.4 (21.3–235.7) pg/mL versus 13.6 (0.0–31.6) pg/mL (*P* = 0.007) for RpfB, 45.0 (25.9–115.8) pg/mL versus 24.9 (0.0–30.4) pg/mL (*P* = 0.03) for RpfC, and 138.0 (67.5–340.5) pg/mL versus 49.3 (16.3–105.1) pg/mL (*P* = 0.004) for RpfD (see Fig. 2). Purified protein derivative induced a higher IFN-γ response in infected children (medium (IQR) 1078.9 (113.1–3937.8) pg/mL versus 92.7 (3.8–350.1) pg/mL, *P* = 0.01).

### Performance of RpfB and D in an IGRA-based test for screening MTBC infection

**Fig. 3:**
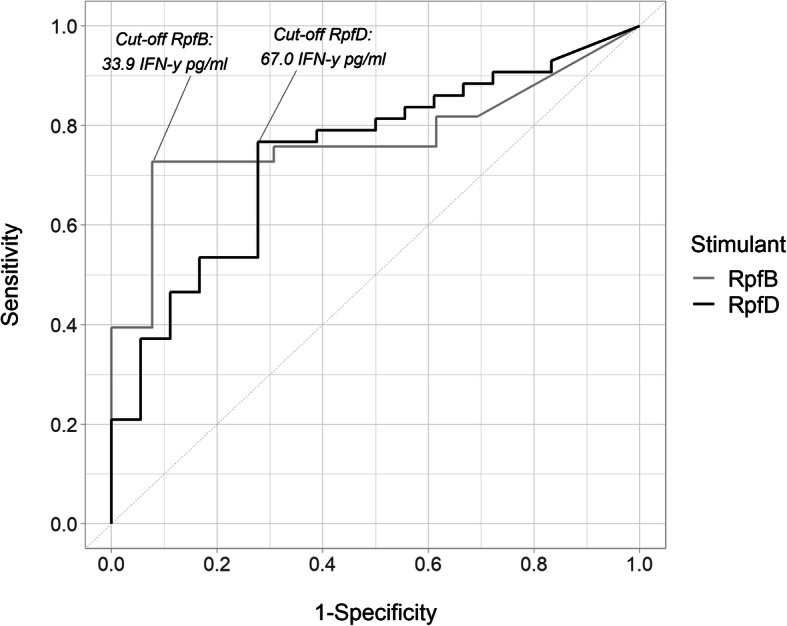
ROC-curves for RpfB and RpfD in an IGRA-based screening for MTBC infection. IGRA+ participants were classified as infected and IGRA- participants were classified as uninfected. AUC’s for RpfB and D are 0.77 and 0.74, respectively. Cut-off values for IFN-γ levels were based on the Youden Index. For RpfB, a cut-off at 33.9 pg/mL led to 73% sensitivity and 92% specificity. For RpfD, a cut-off of 67.0 pg/mL led to 77% sensitivity and 72% specificity. ROC: Receiver operating curve; Rpf: Resuscitation promoting factor; IGRA (interferon gamma release assay); AUC: area under the curve

In our quest for a suitable alternative screening antigen, RpfB and D were found to induce the most differential IFN-γ response (*P* < 0.01; Fig. 2). This result motivated our decision to further analyse their discriminatory capacity using ROC-curves. The area under the curves (AUC) were 0.77 and 0.74 for RpfB and RpfD, respectively (Fig. 3). Based on the Youden Index, a cut-off at 33.9 pg/mL for RpfB-specific IFN-γ levels was derived, leading to 73% (24/33) sensitivity and 92% (12/13) specificity (Fig. 3). Similarly, for RpfD, a cut-off of 67.0 pg/mL led to 77% (33/43) sensitivity and 72% (13/18) specificity (Fig. 3). For the TST, these figures were 65% (22/34) sensitivity and 75% (12/16) specificity (Fig. 1).

In contrast, when using TST results as standard only the stimulations with EC-fusion protein and PPD induced a significantly different IFN-γ response between infected and uninfected groups (*P* = 0.004 and 0.003 respectively [see Additional file 1, Supplementary Figure 4]).

### IFN-γ response to RpfB and D in MTBC infected compared to diseased children

Since RpfB and D responses could discriminate between infected and uninfected children, we evaluated the diagnostic performance of TB disease. Resuscitation promoting factor B and D induced a significantly higher IFN-γ response in infected compared to TB diseased children (medium (IQR) 47.1 (7.1–129.8) pg/mL versus 9.1 (0.0–75.3) pg/mL, *P* = 0.02 and 91.1 (40.9–284.5) pg/mL versus 60.1 (11.0–114.2) pg/mL, *P* = 0.03) respectively (Fig. 2). EC-fusion protein induced higher IFN-γ responses in infected compared to TB diseased children (*P* = 0.0002), but no significant difference was obtained with PPD stimulation between these groups (Fig. 2). There was no significant difference in IFN-γ response between uninfected and TB diseased children for any of the Rpf antigens, EC or PPD (Fig. 2).

### Background IFN-γ response in TB diseased children compared to children without TB disease

In general, TB diseased children had significantly higher background IFN-γ responses (whole blood stimulated with medium alone) compared to the other children without TB disease (irrespective of their infection status) (medium (IQR) 52.6 (42.0–78.4) pg/mL versus 18.6 (0.0–41.7) pg/mL, *P* = 0.0003) (Fig. 2). Using an ROC-curve, we further analysed the medium background response as a screening and/or diagnosis tool in our study setting. The AUC was 0.77, and at optimal cut-off 33.3 mg/mL (based on the Youden Index), sensitivity and specificity were 92.9 and 63.1%, respectively [see Additional file 1, Supplementary Figure 5].

## Discussion

We found that Rpf-specific IFN-γ responses are associated with MTBC infection status in household-exposed children in The Gambia. This suggests that Rpf are attractive antigens to consider for childhood TB screening and diagnostics. Screening for infection and diagnosing TB disease represent core challenges in paediatric TB, particularly in low- and middle-income countries, where TB prevalence is high and other paediatric illnesses with TB-resembling symptoms are common. Given the worldwide shortage of PPD for tuberculin skin testing and its inherent lack of sensitivity and specificity [25], it is essential to develop new methods for MTBC infection screening, particularly for the high-risk group of household-exposed children of infectious adult TB patients, as recommended by the WHO.

We stimulated children’s venous blood samples in vitro with Rpf antigens and showed that RpfB and D induce lower IFN-γ responses in uninfected children compared to infected children. In our study population, RpfB and RpfD -based IGRAs identified infection with higher sensitivity than a TST. This is in line with previous studies in adults. In a multi-side study in adults including The Gambia, Sutherland et al showed that soluble IFN-γ responses after 7 days whole blood stimulation with RpfA and RpfE but not RpfB were higher in TST+ than TST- participants without TB disease [14]. Similarly, Huang et al reported that RpfA and RpfD induced higher IFN-γ responses in “latent tuberculosis infected” (LTBI) household adult contacts compared to community exposed infected participants and healthy controls [16]. Commandeur et al also found that RpfA and RpfD induced different immune responses in vitro in infected adults than in healthy controls [26]. There are clear differences in the discriminatory capacity of the different Rpf proteins in the highlighted previous studies in adults. However, RpfA-induced responses consistently differ between infected and uninfected adults across multiple studies. An explanation for the absence of strong discriminatory RpfA-induced IFN-γ responses in our experiments could be given by the experimental set-up. Our study that focussed on exploring the possibility of integrating Rpf in TB screening, investigated a short-term stimulation of ~ 20 h, contrary to previous studies, which used several days stimulation and added costimulatory molecules such as CD28 and CD49d. In addition, the participants’ younger age likely affected our observed results. Because of the age-dependent maturation of the immune system, children’s IFN-γ production tends to be lower and the contribution of T-cells producing IFN-γ is thought to be less compared to adults [4, 27, 28].

We additionally explored whether Rpf could discriminate between infected and TB diseased children. Resuscitation promoting factor B and D also induced a significantly higher IFN-γ response in infected children compared to TB diseased children. Similar results were previously observed in adults [14, 16, 29]. In vitro*,* RpfA-, B-, D-, and E-specific IFN-γ producing CD4^+^ T-cells are less abundant in TB diseased patients compared to infected individuals [29, 30]. These observations support the hypothesis that a Rpf-specific IFN-γ response can discriminate infected individuals from TB diseased cases. In our study population, Rpf responses did not differ between those with TB disease and those without TB disease (irrespective of infection status), due to the similar Rpf response in children with TB disease compared to uninfected children. Thus, our study does not show discriminatory power of Rpf for TB disease per se, it only shows a difference between children with infection and TB disease. This difference further confirms the pattern of Rpf production during infection, which specifically increase as the bacteria transit from latency to active replication associated with progression to TB disease [15, 16, 26]. With respect to the diagnosis of TB disease, Rpf may only be useful when the infection status of the patient is already ascertained. However, IFN-γ responses to Rpf might indicate whether individuals are at risk of developing TB disease in the near future following infection. Generally, the background IFN-γ production was significantly higher in TB diseased children compared to children without TB disease. The ROC-curve analysis at 33.3 pg/mL cut-off could have identified children with TB disease from children without TB disease with a sensitivity of 92.9%, but with a specificity of 63.1%. Of note, this cut-off is relatively low, in particular when compared to the median (IQR) background IFN-γ level of 23.3 (5.0–37.2) pg/mL. The higher IFN-γ background response in TB diseased children might reflect the disease exacerbation profile that is accompanied by non-specific inflammation and deserves further attention [28, 31].

Our study has some clear limitations regarding infection classification and the study population. We defined MTBC infection by a single test, i.e., IGRA with EC-fusion protein antigen. Although globally recognized as a standard for TB screening, the EC-based IGRA test is known to have lower sensitivity for *M. africanum* infection [6] and in children below 5 years of age [32]. In fact, our study of Rpf-induced IFN-γ production levels in IGRA+ and IGRA- children compares the immunogenicity of the EC and Rpf antigens. Therefore, our strict separation of infected versus uninfected children purely based on IGRA results should be considered with caution, as of course, there is no gold standard for MTBC infection. However, assuming that our IGRA results reflected the participants’ infection status more accurately than the TST results that is confounded by BCG vaccination and exposure to environmental mycobacteria [5], RpfB and D still hold promise for TB screening in children. Another limitation of our study is the modest sample size. This influenced the ROC-analyses, which in turn had impact on the cut-off for IFN-γ response. Another IFN-γ cut-off for RpfB and D would have resulted in a different specificity and sensitivity, which is why we strictly adhered to one method for selecting the ideal cut-off: the Youden Index. Furthermore, all participants were part of a childhood TB contact study, meaning that they had been exposed to an adult smear-positive index TB case. Consequently, no TB-unexposed negative controls were included. Future work should include a larger control group consisting of children with TB-like symptoms, but who do not have TB disease and with confirmed alternative diagnostics. This group of children is the biggest confounder of TB disease diagnosis in children based on clinical symptoms as these symptoms resemble that of other paediatric illnesses occurring in endemic regions [33]. Moreover, our study did not include follow-up data on the progression from infection towards TB disease. Explicitly in the case of Rpf responses, this shift warrants further investigation, because these antigens could offer new possibilities for the prediction of progression to TB disease after exposure [34]. Finally, we could not assess the effect of MTBC strains diversity on the Rpf-specific immune response because there were few bacilli culture positive sample. Future work would benefit from this additional piece of information, to paint a more complete picture of the possibilities and limitations of Rpf in the field of TB.

## Conclusions

Our study shows that immune responses to RpfB and D can discriminate between household TB-exposed infected and uninfected children with higher accuracy than TST screening, with the EC-based IGRA taken as standard for MTBC infection. Additionally, both antigens induce a lower IFN-γ response in children with TB disease compared to infected children and thus warrant further investigation as screening and diagnostic antigens for childhood TB.

## Supplementary information

**Additional file 1 Supplementary Figure 1.** TST induration size versus IGRA with EC-fusion protein. **Supplementary Figure 2.** TST induration size versus age. **Supplementary Figure 3.** IGRA with EC-fusion protein versus age. **Supplementary Figure 4.** IFN-γ response after ex vivo whole blood antigen stimulation using TST as reference standard for MTBC infection. **Supplementary Figure 5.** ROC-curve for background (i.e., unstimulated) IFN-y level for diagnosis of TB disease.

## Data Availability

The datasets and R-scripts used and/or analyzed during the current study are available from the corresponding author on reasonable request.

## Abbreviations

AUC: The area under the curve
BCG: Bacillus calmette-guérin
CFP-10: 10 kDa culture filtrate
EC: ESAT-6/CFP-10-fusion protein
ELISA: Enzyme-linked immunosorbent assay
ESAT-6: 6 kDa early secretory antigenic target
IFN-γ: Interferon gamma
IGRA: Interferon-γ release assays
IQR: Interquartile range
*Mtb*: *Mycobacterium tuberculosis*
*Maf*: *Mycobacterium africanum*
PHA-L: Phytohemagglutinin Antigen-L
PPD: Purified protein derivate
ROC: Receiver operating characteristic
Rpf: Resuscitation promoting factor
TB: Tuberculosis
TST: Tuberculin skin test

## References

[1] Global Tuberculosis Report 2019. *World Health Organization*. (2019). Available online at: https://apps.who.int/iris/bitstream/handle/10665/329368/9789241565714-eng.pdf?ua=1 (17 October 2019).

[2] Frigati L, Maskew M, Workman L, Munro J, Andronikou S, Nicol MP (2015). Clinical predictors of culture-confirmed pulmonary tuberculosis in children in a high tuberculosis and HIV prevalence area. Pediatr Infect Dis J.

[3] Hill PC, Jackson-Sillah D, Fox A, Franken KLMC, Lugos MD, Jeffries DJ (2005). ESAT-6/CFP-10 fusion protein and peptides for optimal diagnosis of mycobacterium tuberculosis infection by ex vivo enzyme-linked immunospot assay in the Gambia. J Clin Microbiol.

[4] Meier NR, Volken T, Geiger M, Heininger U, Tebruegge M, Ritz N (2019). Risk factors for indeterminate interferon-gamma release assay for the diagnosis of tuberculosis in children-a systematic review and meta-analysis. Front Pediatr.

[5] Machingaidze S, Wiysonge CS, Gonzalez-Angulo Y, Hatherill M, Moyo S, Hanekom W (2011). The utility of an interferon gamma release assay for diagnosis of latent tuberculosis infection and disease in children: a systematic review and meta-analysis. Pediatr Infect Dis J.

[6] de Jong BC, Hill PC, Brookes RH, Gagneux S, Jeffries DJ, Otu JK (2006). Mycobacterium africanum elicits an attenuated T cell response to early secreted antigenic target, 6 kDa, in patients with tuberculosis and their household contacts. J Infect Dis.

[7] Gehre F, Antonio M, Otu JK, Sallah N, Secka O, Faal T (2013). Immunogenic Mycobacterium africanum strains associated with ongoing transmission in the Gambia. Emerg Infect Dis.

[8] Rangaka MX, Wilkinson KA, Glynn JR, Ling D, Menzies D, Mwansa-Kambafwile J (2012). Predictive value of interferon-γ release assays for incident active tuberculosis: a systematic review and meta-analysis. Lancet Infect Dis.

[9] Pai M, Denkinger CM, Kik SV, Rangaka MX, Zwerling A, Oxlade O (2014). Gamma interferon release assays for detection of Mycobacterium tuberculosis infection. Clin Microbiol Rev.

[10] Huang W, Qi Y, Diao Y, Yang F, Zha X, Ren C (2014). Use of resuscitation-promoting factor proteins improves the sensitivity of culture-based tuberculosis testing in special samples. Am J Respir Crit Care Med.

[11] Mukamolova GV, Kaprelyants AS, Young DI, Young M, Kell DB (1998). A bacterial cytokine. Proc Natl Acad Sci U S A.

[12] Mukamolova GV, Turapov OA, Young DI, Kaprelyants AS, Kell DB, Young M (2002). A family of autocrine growth factors in Mycobacterium tuberculosis. Mol Microbiol.

[13] Machowski EE, Senzani S, Ealand C, Kana BD (2014). Comparative genomics for mycobacterial peptidoglycan remodelling enzymes reveals extensive genetic multiplicity. BMC Microbiol.

[14] Sutherland JS, Lalor MK, Black GF, Ambrose LR, Loxton AG, Chegou NN (2013). Analysis of host responses to Mycobacterium tuberculosis antigens in a multi-site study of subjects with different TB and HIV infection states in sub-Saharan Africa. PLoS One.

[15] Arroyo L, Marín D, Franken KLMC, Ottenhoff THM, Barrera LF (2018). Potential of DosR and Rpf antigens from Mycobacterium tuberculosis to discriminate between latent and active tuberculosis in a tuberculosis endemic population of Medellin Colombia. BMC Infect Dis.

[16] Huang W, Qi Y, Ren C, Wen H, Franken KL, Ottenhoff TH (2013). Interferon-gamma responses to Mycobacterium tuberculosis Rpf proteins in contact investigation. Tuberc.

[17] Egere U, Togun T, Sillah A, Mendy F, Otu J, Hoelscher M (2017). Identifying children with tuberculosis among household contacts in the Gambia. Int J Tuberc Lung Dis.

[18] Stop TB Partnership Childhood TB Subgroup. Chapter 1: introduction and diagnosis of tuberculosis in children. Int J Tuberc Lung Dis. 2006;10:1091–1097. 17044200

[19] Franken KL, Hiemstra HS, van Meijgaarden KE, Subronto Y, den Hartigh J, Ottenhoff TH (2000). Purification of his-tagged proteins by immobilized chelate affinity chromatography: the benefits from the use of organic solvent. Protein Expr Purif.

[20] Sutherland JS, de Jong BC, Jeffries DJ, Adetifa IM, Ota MOC (2010). Production of TNF-alpha, IL-12(p40) and IL-17 can discriminate between active TB disease and latent infection in a west African cohort. PLoS One.

[21] Togun TO, Egere U, Gomez MP, Sillah AK, Daramy M, Tientcheu LD (2016). No added value of interferon-gamma release to a prediction model for childhood tuberculosis. Eur Respir J.

[22] Mendy J, Jarju S, Heslop R, Bojang AL, Kampmann B, Sutherland JS (2018). Changes in Mycobacterium tuberculosis-specific immunity with influenza co-infection at time of TB diagnosis. Front Immunol.

[23] Kassa D, Ran L, Geberemeskel W, Tebeje M, Alemu A, Selase A (2012). Analysis of immune responses against a wide range of Mycobacterium tuberculosis antigens in patients with active pulmonary tuberculosis. Clin Vaccine Immunol.

[24] Ruopp MD, Perkins NJ, Whitcomb BW, Schisterman EF (2008). Youden index and optimal cut-point estimated from observations affected by a lower limit of detection. Biom J.

[25] Tebruegge M, Bogyi M, Soriano-Arandes A, Kampmann B (2014). Shortage of purified protein derivative for tuberculosis testing. Lancet.

[26] Commandeur S, van Meijgaarden KE, Lin MY, Franken KL, Friggen AH, Drijfhout JW (2011). Identification of human T-cell responses to Mycobacterium tuberculosis resuscitation-promoting factors in long-term latently infected individuals. Clin Vaccine Immunol.

[27] Kumar NP, Anuradha R, Suresh R, Ganesh R, Shankar J, Kumaraswami V (2011). Suppressed type 1, type 2, and type 17 cytokine responses in active tuberculosis in children. Clin Vaccine Immunol.

[28] Basu Roy R, Whittaker E, Seddon JA, Kampmann B (2019). Tuberculosis susceptibility and protection in children. Lancet Infect Dis.

[29] Riano F, Arroyo L, Paris S, Rojas M, Friggen AH, van Meijgaarden KE (2012). T cell responses to DosR and Rpf proteins in actively and latently infected individuals from Colombia. Tuberc..

[30] Schuck SD, Mueller H, Kunitz F, Neher A, Hoffmann H, Franken KL (2009). Identification of T-cell antigens specific for latent mycobacterium tuberculosis infection. PLoS One.

[31] Tientcheu LD, Haks MC, Agbla SC, Sutherland JS, Adetifa IM, Donkor S (2016). Host immune responses differ between M africanum- and M tuberculosis-infected patients following standard anti-tuberculosis treatment. PLoS Negl Trop Dis.

[32] Ferrara G, Losi M, D’Amico R, Roversi P, Piro R, Meacci M (2006). Use in routine clinical practice of two commercial blood tests for diagnosis of infection with Mycobacterium tuberculosis: a prospective study. Lancet.

[33] Marais BJ, Gie RP, Hesseling AC, Schaaf HS, Lombard C, Enarson DA (2006). A refined symptom-based approach to diagnose pulmonary tuberculosis in children. Pediatrics.

[34] Arroyo L, Rojas M, Franken KL, Ottenhoff TH, Barrera LF (2016). Multifunctional T cell response to DosR and Rpf antigens is associated with protection in long-term Mycobacterium tuberculosis-infected individuals in Colombia. Clin Vaccine Immunol.

