# Supplementary material for: Use of resuscitation promoting factors to screen for tuberculosis infection in household-exposed children in The Gambia
Source: BMC Infect Dis. 2020 Jul 2;20:469. doi: 10.1186/s12879-020-05194-1 (PMC7330976; doi:10.1186/s12879-020-05194-1)
Supplement: Supplementary file 1 — Additional file 1 Supplementary Figure 1. TST induration size versus IGRA with EC-fusion protein. Supplementary Figure 2. TST induration size versus age. Supplementary Figure 3. IGRA with EC-fusion protein versus age. Supplementary Figure 4. IFN-γ response after ex vivo whole blood antigen stimulation using TST as reference standard for MTBC infection. Supplementary Figure 5. ROC-curve for background (i.e., unstimulated) IFN-y level for diagnosis of TB disease. [file 12879_2020_5194_MOESM1_ESM.pdf]

Use of Resuscitation promoting factors to screen for tuberculosis infection in household-exposed children in The Gambia

Additional File 1

W van Loon[1,*] , MP Gomez[2], D Jobe[2], KLMC Franken[3], THM Ottenhoff[3], M Coninx[2], L Kestens[4,5], JS Sutherland[2], B Kampmann[2,6], LD Tientcheu[2,7,8,*]

[1] Institute of Tropical Medicine and International Health, Charité-University Medicine Berlin, Berlin, Germany.

[2] Vaccines and Immunity Theme, MRC Unit The Gambia at London School of Hygiene & Tropical Medicine, Fajara, The Gambia.

[3] Department of Infectious Diseases, Leiden University Medical Center, Leiden, The Netherlands.

[4] Immunology Department, Institute of Tropical Medicine Antwerp, Antwerp, Belgium.

[5] Faculty of Pharmaceutical, Biomedical and Veterinary Sciences, University of Antwerp, Antwerp, Belgium.

[6] The Vaccine Centre, Faculty of Infectious & Tropical Diseases, London School of Hygiene & Tropical Medicine, London, UK

[7] Department of Biochemistry, Faculty of Science, University of Yaoundé 1, Yaoundé, Cameroon

[8] Department of Infection Biology, Faculty of Infectious and Tropical Diseases, London School of Hygiene & Tropical Medicine, London, UK

*** Correspondence:**

Welmoed van Loon; welmoed.vanloon@gmail.com

Dr. Leopold D. Tientcheu; ltientcheu@mrc.gm

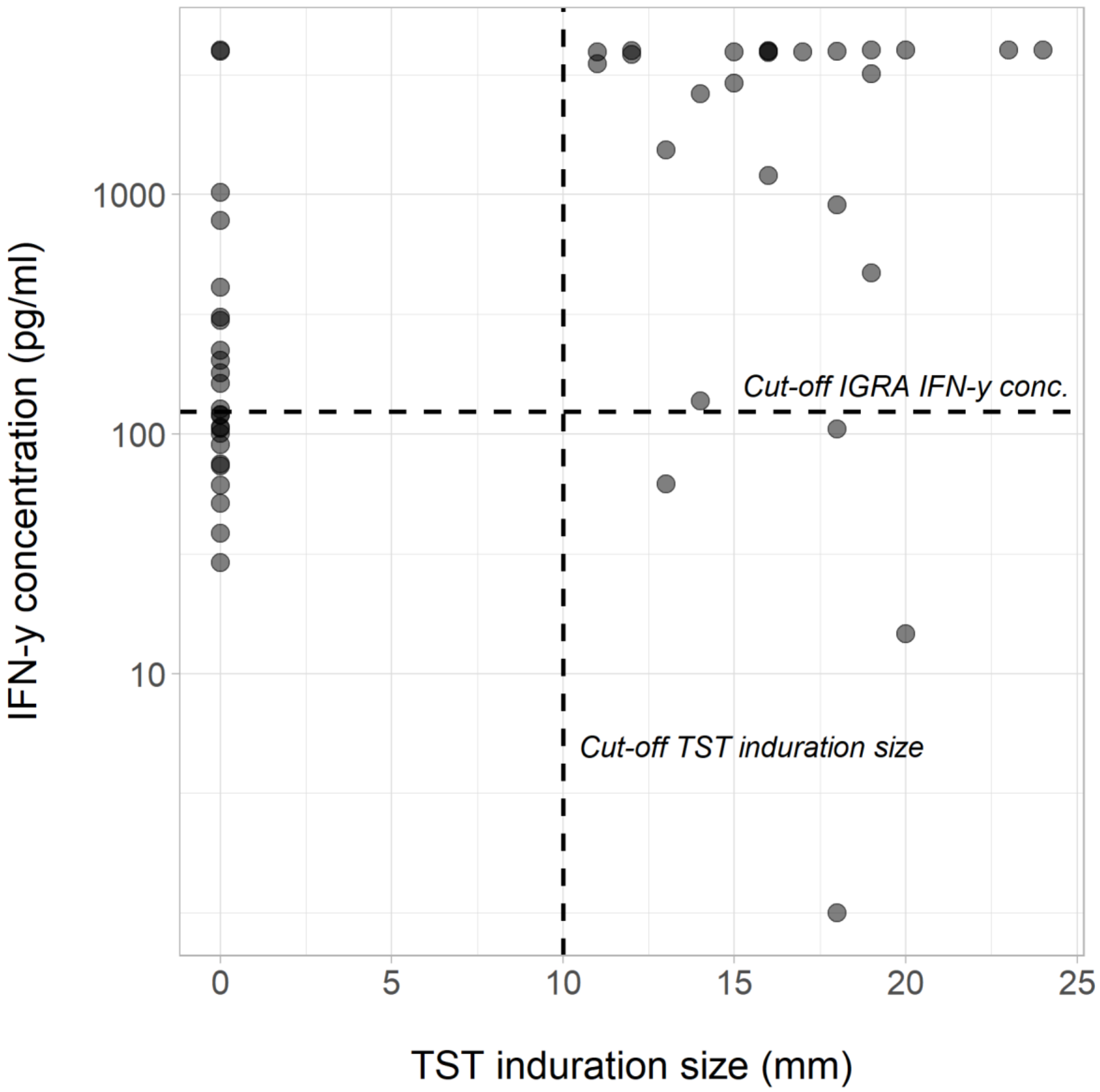

Supplementary Figure 1. TST induration size *versus* IGRA with EC-fusion protein. IFN-γ concentrations were corrected for the background signal (value of unstimulated IFN-γ level subtracted) and all values above 4000 were set to 4000 pg/mL. TST induration size and IFN-γ were significantly correlated (Spearman's ρ 0.4; P = 0.0006). TST = Tuberculin skin test, IGRA = IFN-γ release assay, EC = ESAT-6/CFP-10.

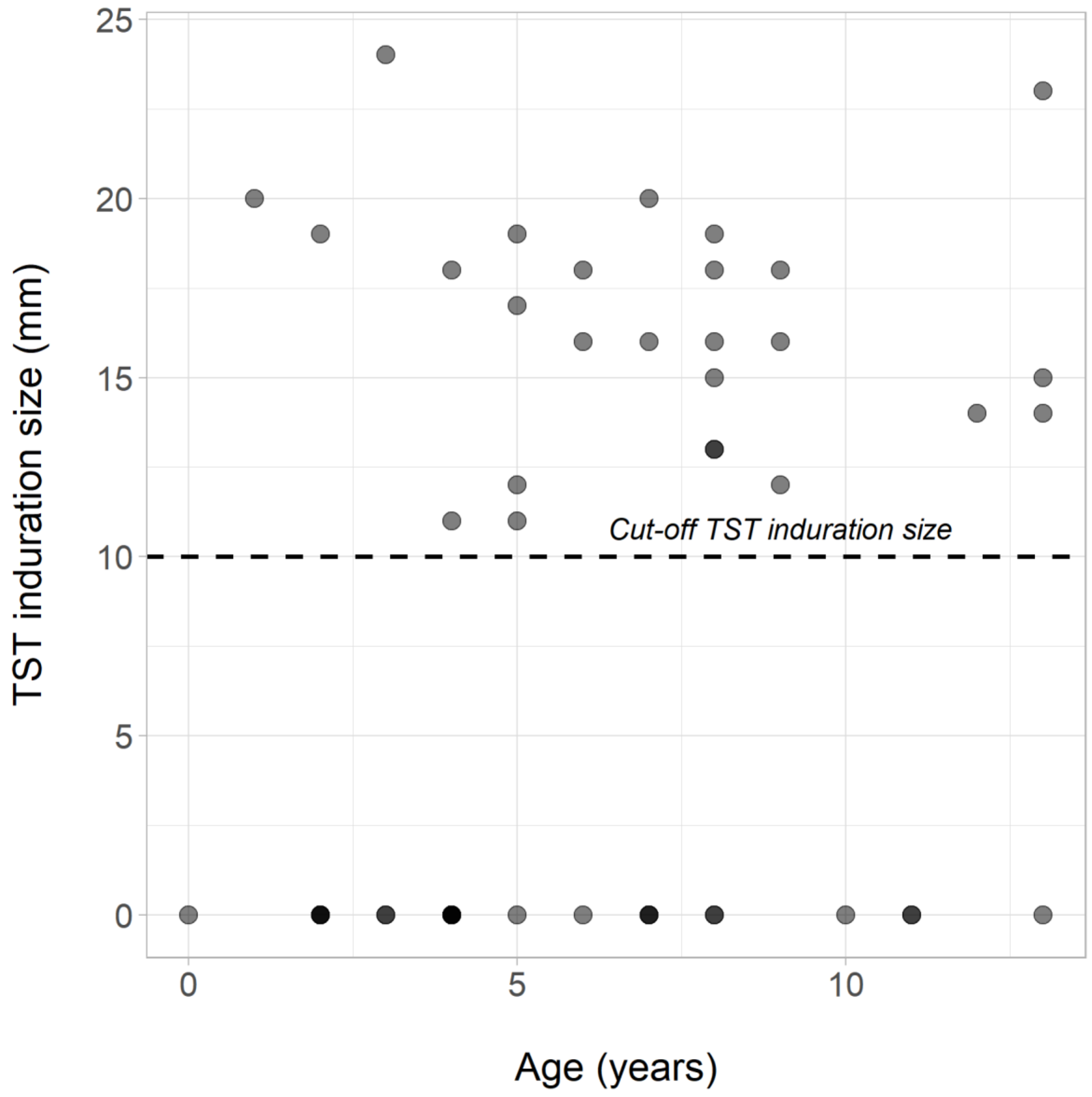

Supplementary Figure 2. TST induration size *versus* age.

TST+ children were significantly older than TST- children (median 7.5 (IQR 5.0-8.8) years versus 4.0 (IQR 3.0-7.3) years; P = 0.05). TST = Tuberculin skin test.

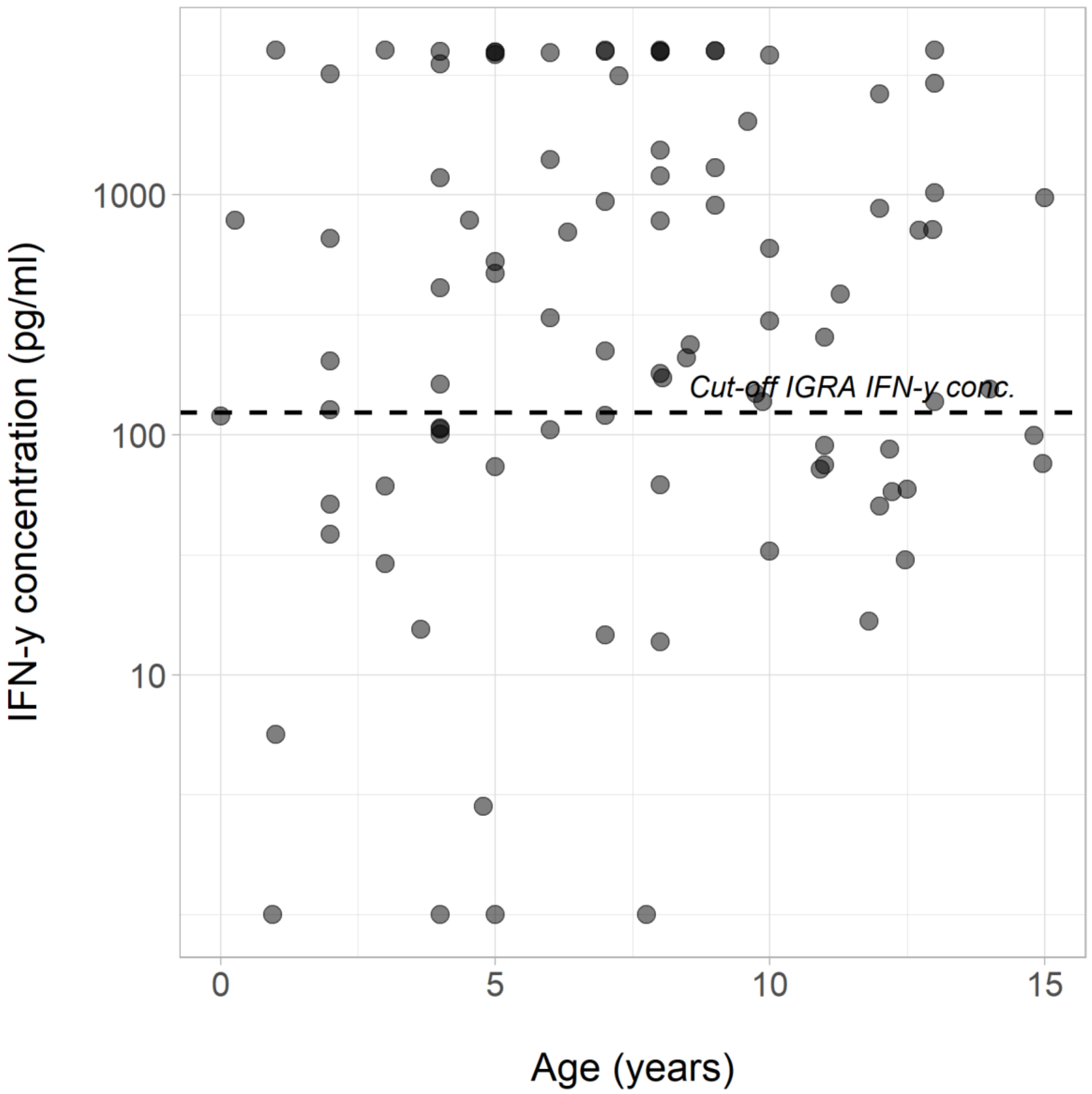

Supplementary Figure 3. IGRA with EC-fusion protein *versus* age.

IGRA+ children were tended to be older than IGRA- children (median 8.0 (IQR 5.0-10.0) years versus median 4.5 (IQR 3.0-8.0) years; P = 0.06). IGRA = IFN-γ release assay, EC = ESAT-6/CFP-10.

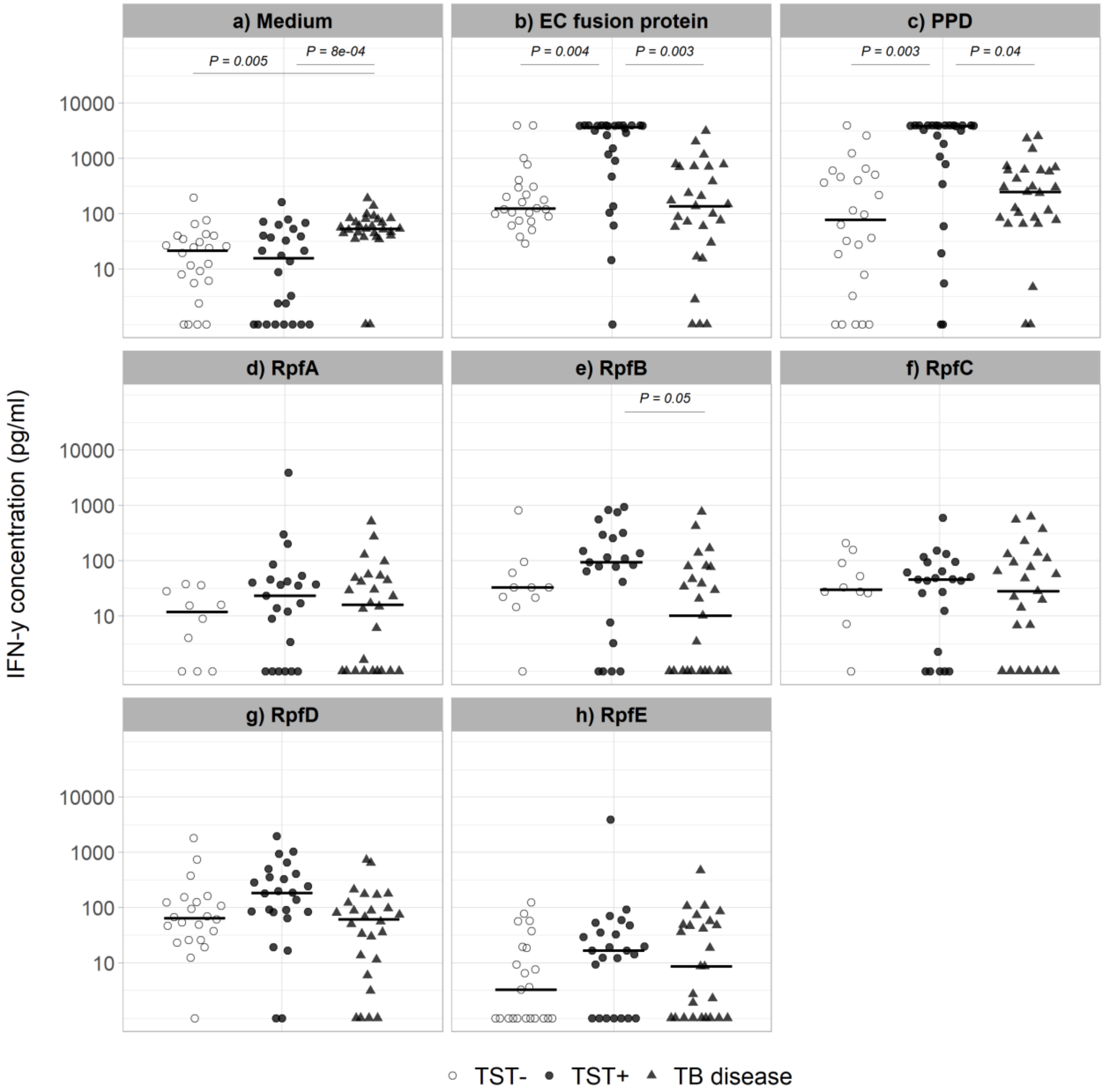

Supplementary Figure 4. IFN-γ response after *ex vivo* whole blood antigen stimulation using TST as reference standard for MTBC infection.

TST+ participants (filled circle) were classified as infected and TST- participants (open circle) were classified as uninfected. The first panel (a) "medium" depicts the results of the unstimulated assays, i.e., the background level. The other panels (b – h) represent the respective antigen-stimulated assays. Solid lines: median IFN- γ values per infection category. IFN-γ concentrations were corrected for the background signal (value of unstimulated IFN-γ level subtracted), except for the results in the first panel "medium". All values above 4000 were set to 4000 pg/mL. P-values below 0.05 indicated. *P*-values were derived from logistic regression models including possible confounders age and sex, after log-transformation of the IFN-γ variable. EC = Esat-6/CFP-10, PPD = Purified protein derivative, Rpf = Resuscitation promoting factor, TST= Tuberculin skin test.

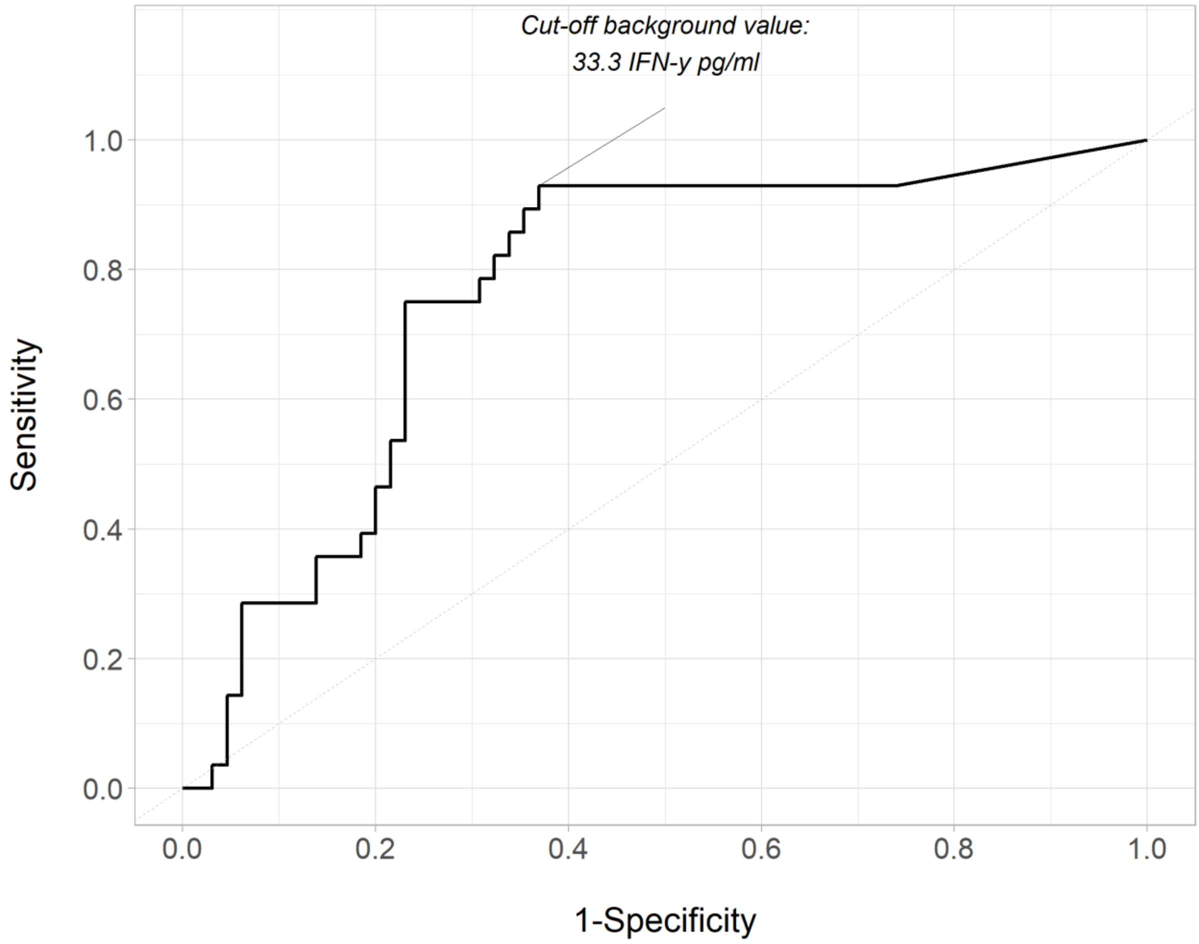

Supplementary Figure 5. ROC-curve for background (*i.e.*, unstimulated) IFN-y level for diagnosis of TB disease.

TB disease was diagnosed by mycobacterial confirmation and/or clinical diagnosis. Participants without TB disease could still be infected or symptomatic. AUC is 0.77 Cut-off value for IFN-γ level was based on the Youden Index. The cut-off was set to 33.3 and led to 92.9% sensitivity and 63.1% specificity. ROC = Receiver operating curve, AUC = area under the curve.
